# Independent Biaxial Scanning Light Detection and Ranging System Based on Coded Laser Pulses without Idle Listening Time

**DOI:** 10.3390/s18092943

**Published:** 2018-09-04

**Authors:** Gunzung Kim, Yongwan Park

**Affiliations:** Department of Information and Communication Engineering, Yeungnam University, 280 Daehak-Ro, Gyeongsan, Gyeongbuk 38541, Korea; gzkim@yu.ac.kr

**Keywords:** LIDAR, time-of-flight, idle listening time, range ambiguity, IM/DD OCDMA, free-space optical communication

## Abstract

The goal of light detection and ranging (LIDAR) systems is to achieve high-resolution three-dimensional distance images with high refresh rates and long distances. In scanning LIDAR systems, an idle listening time between pulse transmission and reception is a significant obstacle to accomplishing this goal. We apply intensity-modulated direct detection (IM/DD) optical code division multiple access (OCDMA) using nonreturn-to-zero on-off keying to eliminate the idle listening time in scanning LIDAR systems. The transmitter records time information while emitting a coded laser pulse in the measurement angle derived from the pixel information as the measurement direction. The receiver extracts and decodes the reflected laser pulses and estimates the distance to the target using time-of-flight until the pulse is received after being transmitted. Also, we rely on a series of pulses and eliminate alien pulses via several detection decision steps to enhance the robustness of the decision result. We built a prototype system and evaluated its performance by measuring black matte and white paper walls and assessing object detection by measuring a watering can in front of the black matte paper wall. This LIDAR system eliminated both shot and background noises in the reception process and measured greater distances with improvements in accuracy and precision.

## 1. Introduction

Mobile scanning light detection and ranging (LIDAR) is a critical component of autonomous vehicles which is used to recognize pedestrians [1], street lighting poles [2], and roads [3,4] by processing point cloud data [5,6,7,8,9,10]. All mobile scanning LIDAR systems measure distance using azimuth and elevation information. Some scanning LIDAR systems can also measure reflective intensities and velocities. LIDAR enhances object detection and collision avoidance while traveling at highway speeds by gathering billions of data points in real time. High-resolution and high-speed mobile LIDAR systems are essential for performing this task at speeds greater than 50 km/h [11]. The faster the vehicle is traveling, the more quickly data are needed for the safe operation of the vehicle.

LIDAR operates by emitting a laser pulse and the time-of-flight (ToF) need to travel from the transmitter to a target object and back [12,13,14,15,16,17]. The main drawback of pulsed scanning LIDAR is that its maximum measurable range is proportional to the maximum pulse repetition period, and high-angular-resolution scanning is only possible at low revolutions per second. Table 1 shows a simple comparison of two representative commercial scanning LIDAR systems: SICK LMS511 [18] and Velodyne HDL-64E [19]. Each product has the maximum number of measurement points per second and operates within a specific distribution of angular resolution, measurement points, and revolutions per second [16,19]. The number of revolutions per second decreases with increasing angular resolution, as does the number of measurement points with a horizontal field of view (FoV). We consider the design of a surround-view-capable pulsed scanning LIDAR that can measure a target at 100 m, with a 360° horizontal FoV, 20° vertical FoV, and 0.2° angular resolution at 20 revolutions per second. A 0.277 μs pulse repetition period is needed to meet the design goals. However, we cannot accomplish these design requirements with a 1.5 MHz light source and photodetector; therefore, we must either compromise the design requirements or invent a different approach. Increasing the maximum measurable range and the number of measurement points at the same time is challenging because the operation of the direct ToF method depends on optical characteristics such as the speed of light.

The key performance indicators for LIDAR are the maximum range, range resolution, positional precision and accuracy, angular resolution, horizontal and vertical FoV, frame refresh rate, and transmit power [20]. These indicators are mutually related; therefore, if we improve one, the others become weaker. Pulsed scanning LIDAR calculates the distance from ToF data when a pulse width reflected intensity greater than a detection threshold is received after transmitting a laser pulse. Therefore, after LIDAR pulse transmission, the idle listening time for reception necessarily increases in proportion to the maximum measurement range [13,14,15]. The random pattern technique [21,22] and multiple repetition rates [23] are introduced to solve the range ambiguity. The random pattern technique can identify the exact ToF through a correlation between the transmitted and received patterns, and can extend the unambiguous range by increasing the length of the repeated pattern. It is difficult to set a suitable discriminating level to adequately distinguish between the low-intensity reflected pulses in the presence of other high-intensity return pulses. The other method employs pulsed lasers with multiple repetition rates to resolve the range ambiguity. This method can record the different arrival times of the scattered return photons from the non-cooperative target at different repetition rates to determine the measured distance. It cannot resolve the range ambiguity, but it offers a robust and convenient method to decrease the problem.

**Figure 1:**
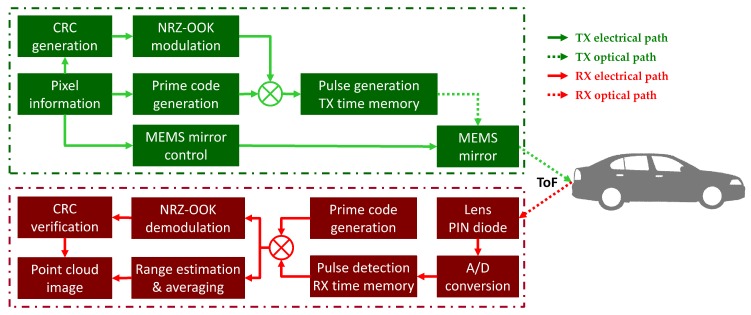
Overall architecture and operation flow of the proposed scanning light detection and ranging (LIDAR) system.

We design an independent biaxial scanning LIDAR system with optical coded pulses to eliminate the idle listening time between the transmitter and the receiver and then build a simple prototype architecture to assess this system [24,25,26]. The prototype uses intensity-modulated direct detection (IM/DD) optical code division multiple access (OCDMA)-coded laser pulses to identify pixel locations and determine the distance to an object. It also employs a two-axis microelectromechanical system (MEMS) mirror to steer the angular direction toward a specific measurement point. In this system, the transmitter and receiver, each consisting of an optical biaxial structure, face forward and operate independently. The transmitter encodes pixel information that is generated according to the measurement point and fires in the bearing direction using the optical modulator and MEMS mirror without waiting to receive the reflected pulse. The receiver receives the signal using a photodiode and analog-to-digital converter (ADC), extracts the pulse through sliding correlation, and decodes it by cross-correlation. We calculate the distance using the ToF between the transmission and the reception of the pulse. We use the cross-correlation value as the received signal strength. The averaged distance of pulses belonging to the same pixel is the pixel’s distance. The sum of the received powers of the pulses is the pixel’s intensity. The maximum range does not affect the system’s operation, and the numbers of revolutions per second and measurement angles are completely independent. The performance goals include a 1 Hz frame refresh rate, an image size of 30 × 30 pixels, and a 10 × 10${}^{\circ }$ FoV. Figure 1 illustrates the overall architecture and operation flow of the proposed scanning LIDAR system.

## 2. LIDAR System Design with Optical Coded Pulses

At each pixel, the proposed LIDAR system generates pixel information to identify the measuring point and emission time. The pixel information is represented with a nine-bit stream, consisting of a leading ‘1,’ a five-bit column identification number (CID), and a three-bit cyclic redundancy check (CRC) checksum [27]. The CID represents the locations of corresponding pixels in each measurement angle and identifies each of the 30 columns from a 30 × 30 range image. The IM/DD OCDMA technique encodes pixel information using a one-dimensional unipolar asynchronous prime sequence code and non-return-to-zero on-off keying (NRZ-OOK) modulation [28,29,30,31,32,33]. Each CID has a distinct binary codeword ($C_{T}$) made up of some number of binary chips, which are regions of constant signal value. Each element ($s_{T,j}$) of the prime sequence code $S_{T}=\left(s_{T,0},s_{T,1},\cdots ,s_{T,i},\cdots ,s_{T,p-1}\right)$ of prime number *p* is determined using $s_{T,j}=Tj\;\left(\mathrm{mod}\;p\right)$, where $s_{T,j}$, *T*, and *j* are all in Galois field GF(p). There are a total of *p* prime sequence codes ($S_{T}$), indexed by T=0,1,⋯,p−1. Each of the *p* prime sequence codes is mapped to a binary codeword ($C_{T}=\left(c_{T,0},c_{T,1},c_{T,2},\cdots ,c_{T,l},\cdots ,c_{T,p^{2}-1}\right)$) of length $p^{2}$, with binary chip $c_{T,j}$ that is determined as follows:

(1) $$c_{T,j}=\left\{\begin{array}{cc} 1\; & \;\mathrm{if}\;l=s_{T,j}+jp\;\mathrm{for}\;j=0,1,\cdots ,p-1\\ 0\; & \;\mathrm{otherwise} \end{array}\right..$$

When the bit has a value of ‘1,’ it is converted to the binary codeword ($C_{T}$). When the bit has a value of ‘0,’ all chips are converted to binary 0. To send pixel information, these $C_{T}$ and equal length binary 0s are concatenated into a codeword sequence. A Gaussian-shaped LIDAR pulse $W_{T}\left[CID\right]\left[n\right]$ is transmitted to each binary chip 1 of $c_{T,j}$ and the time ($TIME_{TX}\left[CID\right]\left[n\right]$) is recorded as follows:

(2) $$W_{T}\left[CID\right]\left[n\right]=\sum_{k=1}^{N}P_{T}\left[t_{k}\right]=\sum_{k=1}^{N}As\left[t_{k}\right]=\sum_{k=1}^{N}\frac{A}{\sigma_{w}\sqrt{2\pi }}e^{-\frac{t_{k}^{2}}{2\sigma_{w}^{2}}}$$

(3) $$TIME_{TX}\left[CID\right]\left[n\right]=current\_time$$

where *n* is the position of binary chip 1 in CID’s binary codewords; $P_{T}$ is the transmitted power in the laser pulse as a function of time; *k* is the time; *N* is the maximum number of time bins in the transmitted pulse; $t_{k}$ is the time of transmission; *A* is the amplitude of the transmitted pulse; and $\sigma_{w}$ is the full width at half-maximum of the Gaussian pulse shape. The transmitter adjusts the angle of the MEMS mirror based on the pixel information, emits and deflects the IM/DD OCDMA-encoded laser pulses in the desired bearing direction, and stores information about the CID and transmission time to calculate the ToF.

The receiver uses a lens to collect the reflected wave and then digitizes the data with the received time using a positive-intrinsic-negative (PIN) photodetector, transimpedance amplifier (TIA), and high-speed ADC. A signal ($W_{R}$) is received, which is a delayed version of the transmitted signal ($W_{T}$) and contains the reflection of the pulse from the object and various kinds of noise and record the time ($TIME_{RX}\left[t_{s}\right]$) as follows:

(4) $$W_{R}\left[t_{s}\right]=\sum_{k=1}^{N}P_{R}\left[t_{k}\right]=\sum_{k=1}^{N}\left(As\left[t_{k}-D\right]+n\left[t_{k}\right]\right)$$

(5) $$TIME_{RX}\left[t_{s}\right]=current\_time$$

where $t_{s}$ is the sampled time; $P_{R}$ is the received power in the laser pulse as a function of time; *D* is a delay factor proportional to the distance to the object; and *n* is noise. A sliding correlation is performed to detect the presence of a Gaussian-shaped LIDAR pulse [32,34,35,36,37,38]. The sliding correlation (SC) measures the similarity between the transmitted signal ($W_{T}$) and the received signal ($W_{R}$):

(6) $$SC=\sum_{m=1}^{N}sc\left[m\right]=\sum_{m=1}^{N}\Theta_{W_{T}W_{R}}=\sum_{m=1}^{N}\sum_{k=1}^{N}P_{T}\left[t_{k}-m\right]P_{R}\left[t_{k}\right]$$

where *m* is a SC variable. After that, a sequence of decisions is made with the SC values. If a SC value (SC) is produced, a constant (*C*), called a threshold, might be chosen and decided for each sample. The decision is then passed to the cross-correlation function with a received waveform (*W*)*R* as an extracted waveform ($W_{E,l}$), the received power in the laser pulse as a function of time ($P_{R}$) as the extracted power ($P_{E,l}$), the sliding correlation (sc[m]) as the peak amplitude of received pulse ($i_{E,l}$), a value of binary 1 as the code element ($c_{E,l}$), and the sampled time ($TIME_{RX}$) as the arrival time of the waveform. The first bit of binary chip sequence of the IM/DD OCDMA-encoded pixel information is always binary chip 1 [29,33]. After an extracted waveform regarded as binary chip 1 is received, the receiver converts continuous waveforms into binary codeword. From the converted binary codeword ($C_{E}=\left(c_{E,0},c_{E,1},\cdots ,c_{E,l},\cdots ,c_{E,p^{2}-1}\right)$), the receiver detects data with the encoded binary codeword $C_{T}$ for CIDs using the aperiodic cross-correlation function shown in Equation (7).

(7) $$\Theta_{C_{T}C_{E}}=\sum_{l=0}^{p^{2}-1}C_{T,l}C_{E,l}$$

where $c_{T,l}$ and $c_{E,l}$ represent the binary chip in the $l^{th}$ positions of $C_{T}$ and $C_{E}$, respectively. The binary codeword is converted into a bit and the ToF dT(l) is calculated if the correlation peak for the code is equal to the prime number (*p*):

(8) $$dT\left(l\right)=TIME_{RX}\left[l\right]-TIME_{TX}\left[CID\right]\left[l\right].$$

The exact target distance is determined by calculating the cross-correlation value between the transmitted and received waveforms using the average square difference function (ASDF) method [39]. The previously extracted waveform is used as the received signal, which is shifted and compared with a fixed portion of the transmitted signal in the estimation window. The position with the highest correlation is considered the exact target location. The ASDF estimator (${\hat{D}}_{ASDF}$) and cross-correlation function (${\hat{R}}_{ASDF}$) are expressed as follows:

(9) $${\hat{D}}_{ASDF}\left(l\right)≐\mathrm{argmin}{\hat{R}}_{ASDF}\left(l,\tau \right)$$

(10) $${\hat{R}}_{ASDF}\left(l,\tau \right)≐\frac{1}{N}\sum_{k=1}^{N}{\left(P_{T}\left[t_{k}\right]-P_{E,l}\left[t_{k}-\tau \right]\right)}^{2}$$

where *N* is the sample number in the estimation window.

According to the central limit theorem, averaging multiple measured results reduces the noise and measurement error in a Gaussian distribution and closes the ground truth value statically [40,41]. The standard deviation provides the root-mean-square width of the Gaussian distribution around the mean, which represents the probability density for the location of the ground truth value. The variance is inversely proportional to the number of samples in the average. Therefore, the more points averaged, the smaller the standard deviation will be from the average and the more accurate the ground truth value will be. An intensity value describes the characteristics of the received signal strength. The total reflected energy of the reflected light pulse is estimated by summing up the peak amplitude of the received pulses ($i_{E,l}$) belonging to the same pixel which allowed the LIDAR system to implement reliability metrics—when detecting objects with stronger reflection signals, the LIDAR system can assign them higher confidence values, thereby enabling more efficient data postprocessing. The target distance (*D*) and the received signal intensity (*I*) are calculated as follows:

(11) $$D=\frac{1}{L}\sum_{n=0}^{L-1}\sum_{l=0}^{p^{2}-1}\left(dT\left(l\right)+{\hat{D}}_{ASDF}\left(l\right)\right)$$

(12) $$I=\sum_{n=0}^{L-1}\sum_{l=0}^{p^{2}-1}i{\left(n\right)}_{E,l}$$

where *l* is the position of pixel information and *L* is the length of pixel information.

The receiver generates the CRC checksum using the CID included in the received bit stream and compares it with that in the received bit stream. If the two CRCs match, the receiver uses the CID to identify the row number and the time at which the received pulses were emitted. A point cloud image is formed whenever the processes are completed for the full set of 30 × 30 pixels.

## 3. Construction of the Prototype LIDAR System

**Figure 2:**
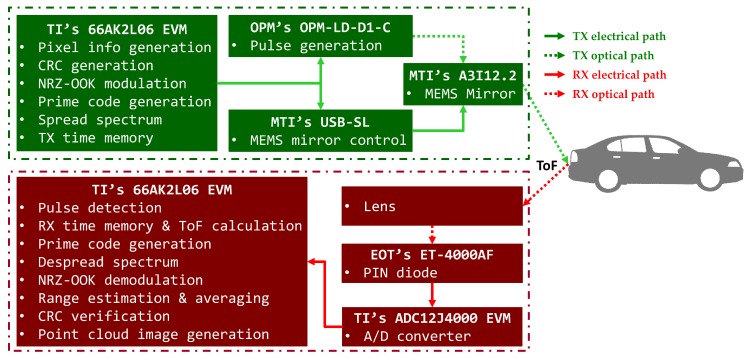
Prototype LIDAR system comprising commercial off-the-shelf products.

A prototype was implemented to validate and assess the proposed scanning LIDAR system. As shown in Figure 2, it comprised commercial off-the-shelf (COTS) products [26], such as an optical modulator module, an amplified photodetector module, a MEMS mirror development kit, an ADC evaluation module, a digital signal processor (DSP) with ARM processor evaluation kit, and a Windows PC.

We used an OPM-LD-D1-C digital high-speed pulsed laser generator as the optical modulator [42]. It is designed for systems that require high-speed transmission and operates at up to 1 GHz, with a peak current of 500 mA and a peak optical power of 250 mW. We used the external trigger as the trigger source and fed an NRZ-OOK modulated stream into it.

**Figure 3:**
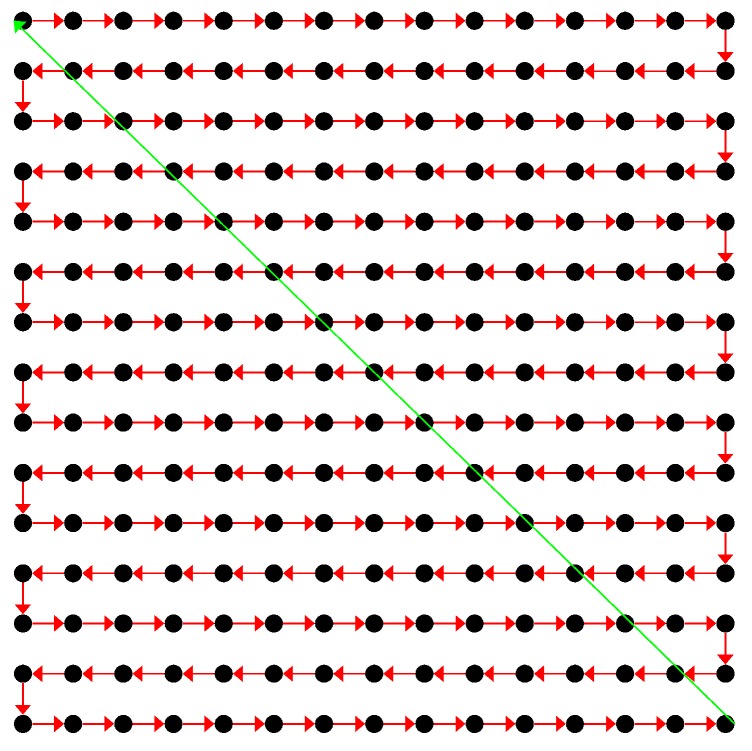
MEMS mirror adapting a bidirectional raster scan pattern.

The coded laser pulses were deflected and steered in the desired measurement angle using a two-axis MEMS mirror from Mirrorcle Technologies, Inc. [43] that was designed and optimized for point-to-point optical beam scanning via a steady-state analog actuation voltage [44]. The aluminum-coated mirror was bonded and had a diameter of 1.2 mm and mechanical tilt angles in a horizontal FoV of −5.0${}^{\circ }$ to 5.0${}^{\circ }$ and in a vertical FoV of −5.0${}^{\circ }$ to 5.0${}^{\circ }$. A universal serial bus MEMS controller connected the MEMS mirror and Windows PC, drove the MEMS mirror via biased differential high analog voltage outputs, and provided a digital output pin DOut0 as a synchronous trigger output at the start of every pixel event. The control software was developed using the C++ software development kit and generated bidirectional raster scan patterns that created uniformly spaced lines along the vertical axis and repeated them on the horizontal axis (Figure 3).

**Figure 4:**
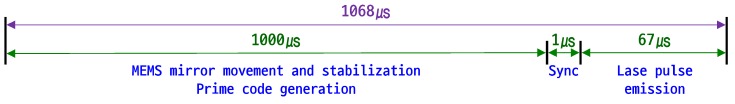
Each pixel has 1068 μs for microelectromechanical system (MEMS) mirror movement, prime code generation, synchronous triggering, and laser pulse emission.

We allocated approximately 1068 μs to each pixel (Figure 4) to acquire a 30 × 30 pixels image at one frame per second. We tilted the MEMS mirror to a measuring point during the first 1000 μs. We then tilted the horizontal axis of the MEMS mirror by 0.345${}^{\circ }$ after measuring a single pixel. Subsequently, we tilted the vertical axis of the MEMS mirror by 0.345${}^{\circ }$ after measuring a single line consisting of 30 pixels. At the same time, the signal processor generated pixel information and was encoded with the IM/DD OCDMA method with a weight of 5 and a length of 25 and waited for the rising edge of the DOut0 pin as a synchronous input trigger. The MEMS mirror controller and driver tilted the MEMS mirror by adjusting the voltage up to 141 V and then sent a synchronization trigger to the DOut0 pin. The signal processor emitted 225 chips of 5 ns pulse width using an optical modulator. It then recorded the CID and the emission time of each chip. The power of the laser pulse emitted in the measurement angle was equal or similar to the maximum accessible emission limit (AEL) of Class 1 laser products [45]. This procedure was repeated for every 30 × 30 pixels group in a frame.

An ET-4000AF from EOT [46,47], which operates at frequencies of up to 9 GHz, was chosen for the high-speed amplified PIN GaAs photodiode equipped with a TIA that senses light levels as low as 100 nW. The frequency response of the laser could be measured when it terminated to 50 Ω at the ADC input port. We selected an ADC12J4000 from Texas Instruments (TI), which is a 12 bit, 4 GHz radio frequency-sampling ADC with a buffered analog input [48,49].

The XEVMK2LX is a full-featured evaluation and development tool for the TI 66AK2L06 SoC with a quadcore 1.2 GHz C66X DSP and a dualcore 1.2 GHz ARM Cortex A15 [50,51]. The data transmission procedure generated a nine-bit stream that was spread and modulated using an IM/DD OCDMA method, emitted using an optical modulator synchronized with the rising edge of the DOut0 pin, and recorded the CID and emission time. The data reception procedure received digitized data, recorded its arrival time, detected a signal, extracted the waveform, estimated range, decoded the binary codeword via the IM/DD OCDMA method, and generated a point cloud image.

## 4. Performance Assessment

### 4.1. Operating Modes and Conditions

**Figure 5:**
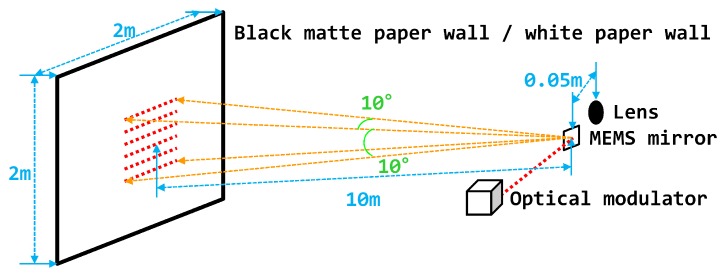
Operating condition and optical structure of the prototype LIDAR system.

We measured the distance and intensity to assess the system’s performance by placing a 2 × 2 m paper wall [52,53,54,55] in front of the prototype LIDAR system (Figure 5). The prototype system was an optical biaxial structure with a 0.05 m distance interval. We operated this LIDAR system in two different modes to assess the performance of the proposed LIDAR system—the legacy mode used only a single pulse for the same pixel, such as other traditional LIDAR systems, whereas the OCDMA mode used all pulses for the same pixel. As presented in Table 2, these two modes had different operating characteristics. During transmission, the legacy mode used 20 nJ for a pulse, whereas the OCDMA mode used 7.8 nJ as its pulse energy to adhere to eye-safety rules for Class 1 lasers. To comply with the AEL, the emission power of the laser pulse was inversely proportional to the pulse width and number. If the width of the pulse became wider or the number of pulses increased, the output power of the pulse had to be reduced. Since the legacy mode and the OCDMA mode use the same pulse width, the output power of the pulse was constrained only by the number of pulses. The legacy mode uses only one pulse at one measurement point, but the OCDMA mode uses several pulses generated through modulation and spreading process, so the pulse power is used relatively low. On the other hand, the OCDMA mode uses 45 pulses compared with one for the legacy mode. For this, the OCDMA mode uses 351 nJ for a per measurement, whereas the legacy mode uses only 20 nJ That suggests that the OCDMA mode uses 17.55 times the energy for each measurement compared to the legacy mode.

The LIDAR’s maximum measured distance depends on the reflectivity of the objects to be detected. Noise can have any value and reaches a detection threshold level. Furthermore, in the presence of the object, the noise and target reflectivity both contribute to the amplitude value. Lowering the detection threshold increases the probability of detection, but also increases the probability that noise exceeds the threshold and causes false alarms [56]. The probability of a false alarm ($P_{FA}$) affects the correctness of the detected return laser pulse and measured distance. In the reception process, the legacy mode relied on single pulse detection and used signal processing with Equations (4), (6) and (10), whereas the OCDMA mode relied on a stream of pulses and eliminated alien pulses via several detection steps with Equations (4)–(12) and the CRC checksum. Because of this difference in the two modes, the legacy mode needed a very low $P_{FA}$ and used a high threshold-to-noise ratio (TNR), but the OCDMA mode used a high $P_{FA}$ and low TNR [57,58]. We selected different detection thresholds for the two modes. The legacy mode used 13.4 dB, while the OCDMA mode used 9.8 dB. The range gate (RG), false alarm rate (FAR), and threshold-to-noise ratio (TNR) shown in Table 2 were calculated as follows:

(13) $$RG=\frac{2(R_{max}-R_{min})}{c}$$

(14) $$FAR=\frac{P_{FA}}{RG}$$

(15) $$TNR=\log_{10}\frac{I_{t}^{2}}{I_{n}^{2}}=\log_{10}\left(-2\ln\left(2\sqrt{3}\tau FAR\right)\right)$$

where RG is a range gate; $R_{max}$ is the desired maximum range; $R_{min}$ is the desired minimum range; FAR is the false alarm rate; $I_{t}$ is the threshold current; $I_{n}$ is the noise current; and τ is the pulse width.

The LIDAR system uses reflected intensity as the peak amplitude of the received pulse for detection. The legacy mode uses one SC, as shown in Equation (6) and Table 2, whereas OCDMA mode uses the sum of all sliding correlation values belonging to the same pixel, as shown in Equation (12) and Table 2. Each pulse used in the summation must have a reflected intensity that can be distinguished from noise to have a valid meaning; thus, the OCDMA mode using the sum of several pulses had a reflected intensity value that was several times higher than that of the legacy mode which used only one pulse.

### 4.2. Pulse Emission Time Interval and Measured Distance

Laser pulses were directly emitted onto the white paper wall in front of the prototype LIDAR to determine the change in distance according to the pulse emission time interval. A laser pulse was emitted at the measurement point to investigate the maximum measurement distance according to the pulse emission time interval. The next pulse was emitted after a predetermined time interval. The pulse emission time interval increased from 5 ns to 100 ns for every 5 ns. The distance to the white paper wall located 10 m from the prototype LIDAR was used as a distance to the corresponding time interval by averaging the distance measured from 1 s to 10 s after starting the distance measurement.

**Figure 6:**
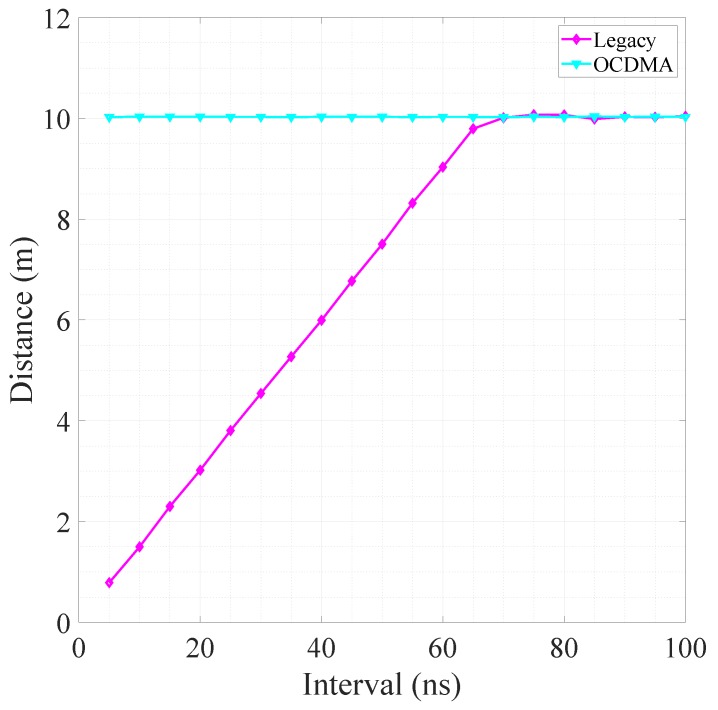
Measured distance for each pulse emission time interval.

Figure 6 shows the relation between the pulse emission time interval and maximum measurement distance in the legacy and OCDMA modes. The pulsed LIDAR calculates the distance by considering the time at which the pulse was received after the emission of the pulse; hence, the maximum measurable range is proportional to the maximum pulse repetition period, and a ToF at least 70 ns is required to measure a distance of 10 m. In the legacy mode, when the pulse emission time interval is less than 70 ns, the pulse emission time interval becomes the ToF, and the maximum measurement distance is thus not 10 m. The ToF increases as the pulse emission time interval increases. The distance to the white paper wall placed 10 m ahead can be accurately measured when the pulse emission time interval is larger than 70 ns. In the OCDMA mode, after receiving the encoded pulses, the pulse release time is obtained together with the measurement point information through pulse decoding. The ToF is determined by the distance to the object (i.e., white paper wall), regardless of the pulse emission time interval. The maximum distance is not affected by the idle listening time between the transmitter and receiver. The distance is accurately measured to the white paper wall located 10 m ahead in a pulse emission time interval of less than 70 ns.

### 4.3. Maximum Distance

**Figure 7:**
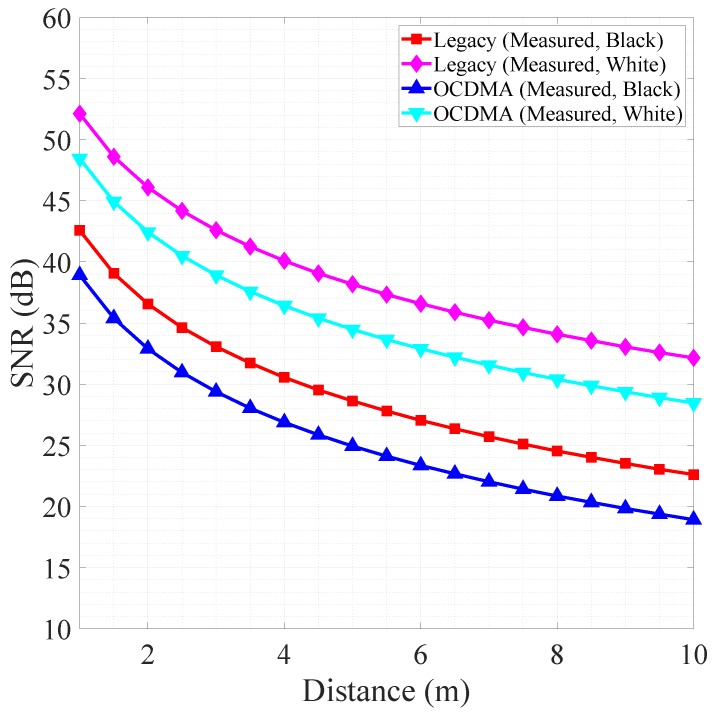
Measured minimum received signal strength every 0.5 m from 1 m to 10 m.

**Figure 8:**
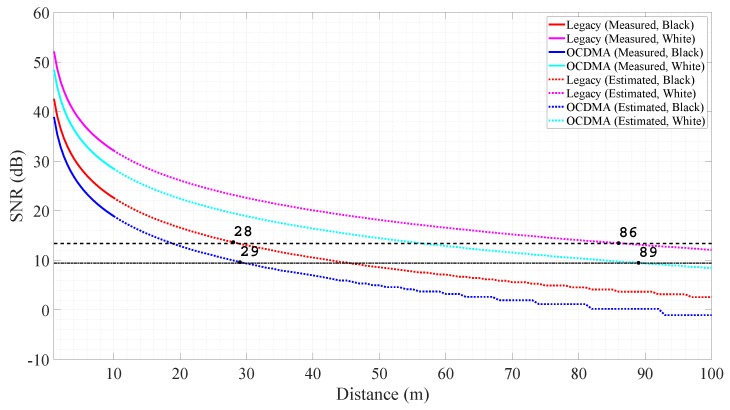
Measured and estimated power with black matte and white paper walls in the legacy and optical code division multiple access (OCDMA) modes.

We measured the distance and intensity for every 0.5 m with 30 × 30 pixels set to assess the system’s maximum distance by alternately placing a 2 × 2 m black matte paper wall and a white paper wall. Figure 7 shows the result of measuring powers every 0.5 m from 1 m to 10 m. This shows the relationship between the received power and the measured distance, as expressed in Equation (16). For an extended Lambertian target, the received signal strength ($P_{det}$) was proportional to the transmitted power ($P_{t}$) and inversely proportional to the square of the distance (*R*) [15,16,59], as shown in Equation (16). The SNR is expressed as the logarithmic expression of the ratio of received power to noise in units of decibels. The OCDMA mode outperformed the legacy mode, regardless of the measured distance or wall color.

(16) $$P_{det}=P_{t}\frac{\pi \tau_{o}\tau_{a}^{2}D_{R}^{2}\rho_{t}}{4R^{2}\theta_{R}}$$

where $\tau_{o}$ is the optics transmission; $\tau_{a}$ is the atmospheric transmission; $D_{R}$ is the receiver aperture diameter; $\rho_{t}$ is the target surface reflectivity; and $\theta_{R}$ is the target surface angular dispersion. In these parameters, the measured distance (*R*) and the target surface reflectivity ($\rho_{t}$) varied with the experimental condition and affected the received signal strength ($P_{det}$). Using the result of the measured power (Figure 7) and the relationship between the received power measured distance, and target surface reflectivity illustrated in Equation (16), we estimated the received power based on the distance. The solid lines in Figure 8 show the measured power result every 0.5 m from 1 m to 10 m, while the dashed lines show the estimated power result every 1 m from 11 m to 100 m. The OCDMA mode used 39% of the energy used in legacy mode for each pulse and 17.55 times the energy for each measurement, hence we could measure 3 m farther (Figure 8). The measurement results of the legacy and OCDMA modes for the black matte and white paper walls are summarized in Table 3. In the OCDMA mode, the intensity was calculated by summing up the peak amplitude of the received pulses belonging to the same pixel shown in Equation (12). The OCDMA mode had an intensity value that was several times higher than that of the legacy mode.

### 4.4. Accuracy and Precision Evaluation

The American Society for Photogrammetry and Remote Sensing (ASPRS) has described the positional standards for digital elevation data [60,61]. In this standard, accuracy is defined as the closeness of a measured value to the ground truth value at a specific confidence level. Precision, related to repeatability, is defined as the approximation with which measurements coincide. In a non-vegetated terrain, the corresponding estimates of accuracy at the 95% confidence level are computed using ASPRS positional accuracy standards such that is approximated by multiplying the root-mean-square-error (RMSE) by 1.96 to estimate the positional accuracy as follows:

(17) $$\mathrm{Accuracy}=\mathrm{RMSE}\times 1.96=\sqrt{\frac{1}{n}\sum_{i=1}^{n}{\left(x_{m,i}-x_{t,i}\right)}^{2}}\times 1.96$$

where $x_{m,i}$ is the coordinate in the specified direction of the *i*th checkpoint in the dataset; $x_{t,i}$ is the coordinate in the specified direction of the *i*th checkpoint in an independent source of higher accuracy; *n* is the number of checkpoints tested; and *i* is an integer ranging from 1 to *n*. The precision is equal to the standard deviation of the measurements. The mean error ($\bar{x}$) and standard deviation ($S_{x}$) are computed as follows:

(18) $$\bar{x}=\frac{1}{n}\sum_{i=1}^{n}x_{i}$$

(19) $$\mathrm{Precision}=S_{x}=\sqrt{\frac{1}{n-1}\sum_{i=1}^{n}{\left(x_{i}-\bar{x}\right)}^{2}}$$

where $x_{i}$ is the *i*th error in the specified direction.

**Figure 9:**
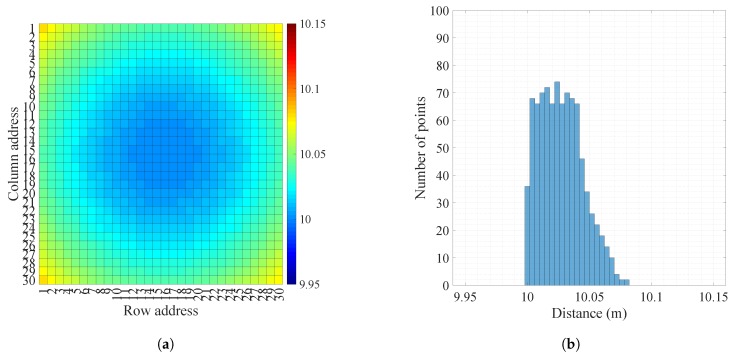

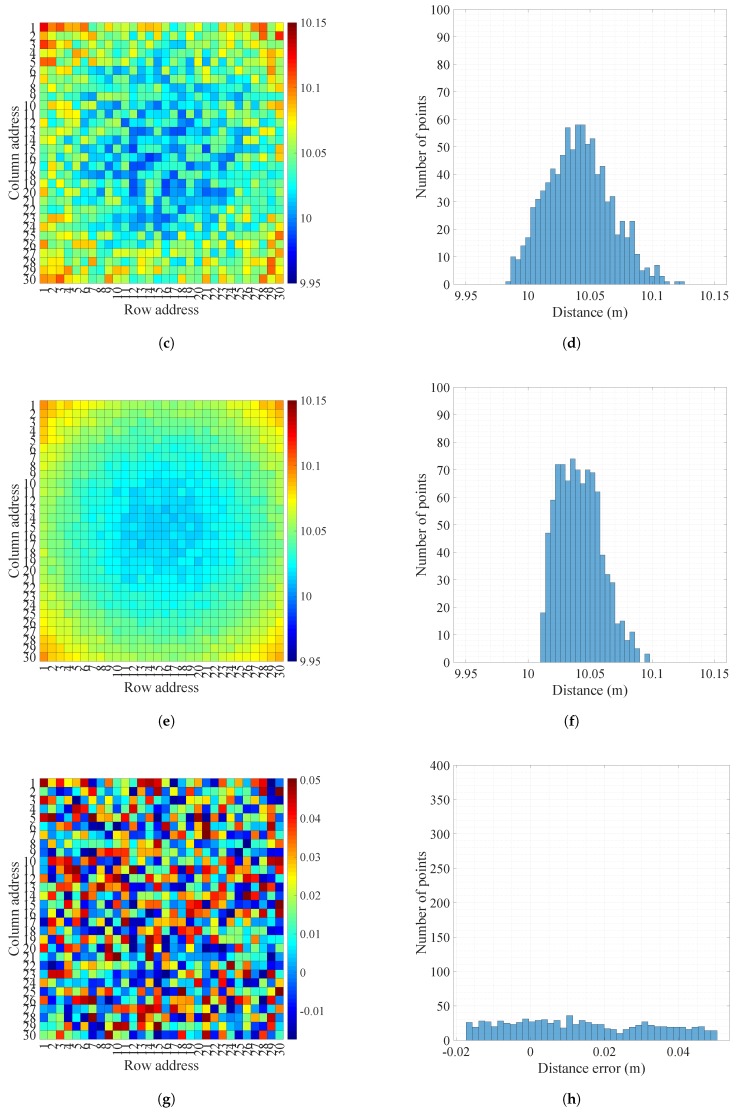

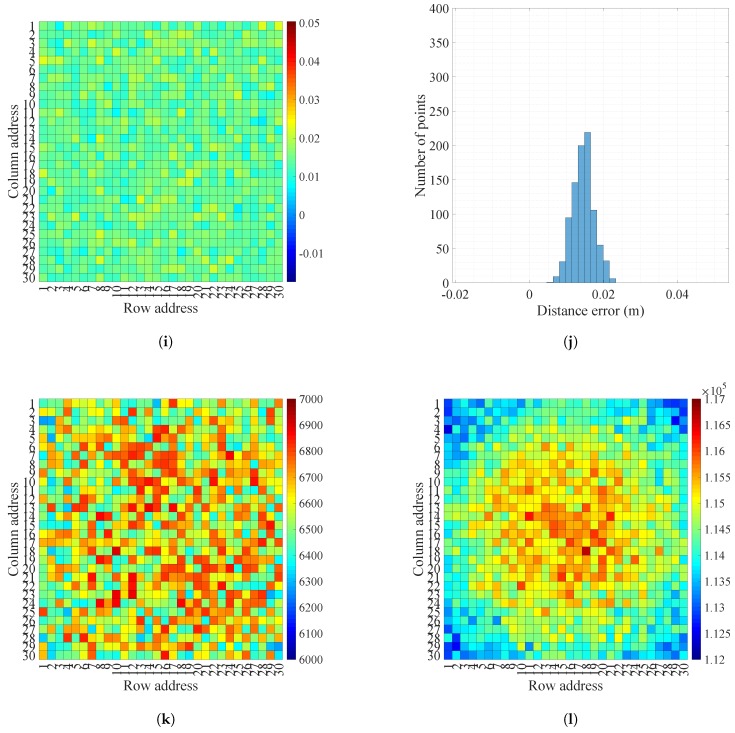
Images of the white paper wall 10 m in front of the LIDAR system: (**a**) distance map and (**b**) histogram of distance in the ground truth; (**c**) distance map and (**d**) histogram of distance in the legacy mode; (**e**) distance map and (**f**) histogram of distance in the OCDMA mode; (**g**) distance error map and (**h**) histogram of distance error in the legacy mode; (**i**) distance error map and (**j**) histogram of distance error in the OCDMA mode; (**k**) intensity map in the legacy mode; (**l**) intensity map in the OCDMA mode.

We calculated the ground truth distance from the geographical relationship between the wall and the prototype LIDAR system. The distance from the center was 10 m. The measuring distance gradually increased as it moved to the edge. It maintained a symmetrical character overall but was slightly biased to the right because of its biased lens. Figure 9 shows a distance map and its histogram of the ground truth distance. Also, it shows the distance maps, histograms of distance, distance error maps, histograms of distance error, and intensity maps measured in the legacy and OCDMA modes.

**Figure 10:**
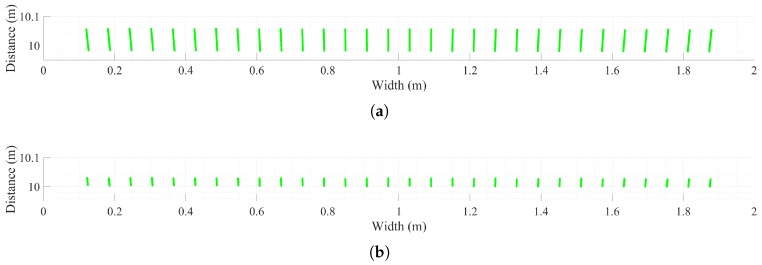
Top-view distance in the legacy (**a**) and OCDMA (**b**) modes.

The distance and intensity values in each map tended to increase with the distance from the center. In the legacy mode, numerous large errors were observed in range estimation. The overall distance measurement result was jagged. However, in the OCDMA mode, only small errors were found in range estimation; thus, the distance map was very similar to the ground truth distance map. The distance error map and the histogram of the legacy and OCDMA modes more clearly showed these characteristics. Figure 10 shows the top-view distances measured in the legacy and OCDMA modes. The distance errors in the legacy mode had longer tails than those in the OCDMA mode for the top-view distance.

Table 4 summarizes the measurement results of the legacy and OCDMA modes. In the legacy mode, the measurement accuracy was 45.8 mm, and the precision was 18.9 mm. In the OCDMA mode, the measurement accuracy improved by 37% to 28.9 mm, while the precision improved by 85% to 2.9 mm. The OCDMA mode exhibited neither shot nor background noise because the receiver implemented the despread spectrum process using a correlation function with its own codeword, and then verified it with the CRC checksum algorithm. Moreover, during reception, the receiver averaged the range of all pulses belonging to the same pixel to reduce range estimation errors. The OCDMA mode used an intensity that was the sum of the reflected signal strengths corresponding to the same pixel position which was 18 times larger than that of the legacy mode.

### 4.5. Sample Object Measurement

**Figure 11:**
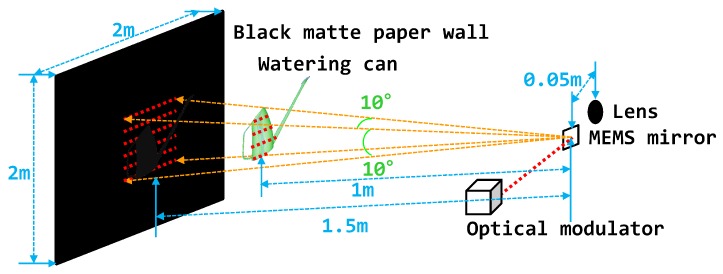
Experimental conditions for the sample object measurement.

**Figure 12:**
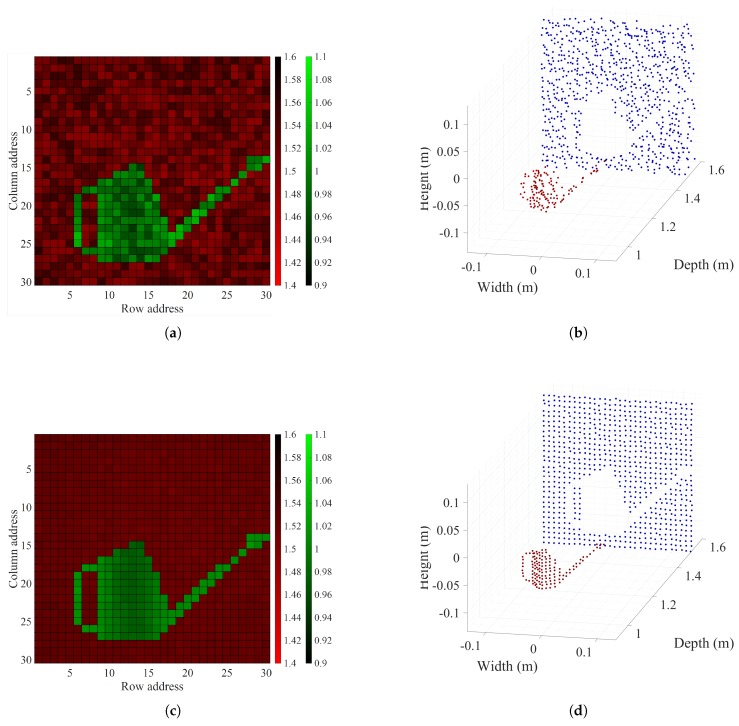
Measured results of the watering can and the black matter paper wall: (**a**) distance map and (**b**) point cloud image in the legacy mode; (**c**) distance map and (**d**) point cloud image in the OCDMA mode.

We placed a 2 × 2 m black matte paper wall and a watering can 1.5 m and 1 m from the proposed LIDAR system to test the sample object measurement of the prototype LIDAR system (Figure 11). The maximum length, height, and width of the watering can were 0.06 m, 0.16 m, and 0.31 m, respectively. In both the distance and intensity images, the outline of the watering can could be differentiated from the black matte paper wall. In Figure 12, the images on the left show the distance map and point cloud image of the measured distances in the legacy mode, whereas those on the right show the results from the OCDMA mode. In all cases, in the legacy mode, noise or defective spots distinctly appeared in the distance map and point cloud image because of considerable errors. In contrast, the OCDMA mode showed relatively few small errors in these images. These results are summarized in Table 5.

## 5. Conclusions

The key performance indicators for LIDAR are the maximum range, range resolution, positional precision and accuracy, angular resolution, horizontal and vertical FoV, and frame refresh rate. These indicators are mutually related; therefore, the others deteriorate if one is improved. In scanning LIDAR systems, the idle listening time between the pulse transmission and reception is a significant obstacle in improving these performance indicators.

We designed and built a prototype to assess a pulsed scanning LIDAR system, designed to encode pixel information in its laser pulses using IM/DD OCDMA to eliminate the idle listening time. The prototype comprises COTS optical components and development kits and achieved a 1 Hz frame refresh rate, an image size of 30 × 30 pixels, and a 10 × 10${}^{\circ }$ FoV. For comparison, the prototype was run in the legacy and OCDMA LIDAR modes. The OCDMA mode averaged multiple measurements and summed all reflected powers to calculate the total reflected energy. Averaging the reduced noise and measurement error and summing enabled us to use stronger and higher confidence values. We assessed the performance of 2 × 2 m black matte and white paper walls and measured a watering can target. In all cases, in the legacy mode, distinct rough spots appeared in the distance map because of many large errors. In the OCDMA mode, few small errors occurred in the range estimation, and the distance map were very similar to the ground truth map. Moreover, both shot and background noise were eliminated by the despread spectrum process and verification with the CRC checksum. The OCDMA mode measured greater distances, with improvements of 37% and 85% in accuracy and precision, respectively. Therefore, we conclude that our proposed LIDAR system is a better alternative to traditional scanning LIDARs.

## Figures and Tables

**Table 1:** Characteristics of two representative LIDAR products on the market.

Product	SICK LMS511			Velodyne HDL-64E		
Bearing mechanism	Deflection of the light using a mirror			Rotation of the light source		
Horizontal FoV	190°			360°		
Vertical FoV	0°			26.8°		
Horizontal angular resolution	0.25°	0.5°	1°	0.0864°	0.1728°	0.3456°
Revolutions per second	25	50	100	5	10	20
Measurements per revolution	761	381	191	266,666	133,333	66,666
Measurements per second	19,025	19,050	19,100	1,333,330	1,333,330	1,333,320

**Table 2:** Operating characteristics of the two modes.

Mode		Legacy	OCDMA
TX	Number of emitted pulses	1	45
	Pulse width (τ)	5 ns	5 ns
	Emitted energy per pulse	20 nJ	7.8 n J
	Emitted energy per measurement	20 nJ	351 nJ
	Number of binary chips	1	225
	Chip emission duration	5 ns	1125 ns
RX	Signal processing method	Equations (4), (6) and (10)	Equations (4)–(12)
	Number of received pulses	1	45
	Maximum desired distance ($R_{max}$)	150 m	150 m
	Range gate (RG)	1 μs	1 μs
	Probability of false alarm ($P_{FA}$)	0.001	0.5
	False alarm rate (FAR)	1000/s	500,000/s
	Threshold-to-noise ratio (TNR)	13.4 dB	9.8 dB

**Table 3:** Summary of the distance and the power for the black matte and white paper walls.

Mode			Legacy	OCDMA
Black matte paper wall	Maximum distance (m)		28	29
	Intensity	1 m	69,844	1,298,513
		10 m	704	12,965
		30 m	78	1444
		90 m	9	160
	SNR (dB)	1 m	42.5754	38.9163
		10 m	22.6099	18.9144
		30 m	13.0551	9.3719
		90 m	3.6766	0.2074
White paper wall	Maximum distance (m)		86	89
	Intensity	1 m	629,636	11,688,489
		10 m	6331	116,857
		30 m	702	12,558
		90 m	78	1446
	SNR (dB)	1 m	52.1250	48.4545
		10m	32.1489	28.4584
		30 m	22.5975	18.9144
		90 m	13.0551	9.3719

**Table 4:** Summary of the distance and intensity measurements for the white paper wall.

Mode		Legacy	OCDMA
Distance (m)	Minimum	9.984	10.0122
	Maximum	10.1258	10.0967
	Accuracy	0.045779	0.028981
	Precision	0.018903	0.0028846
Intensity	Minimum	6275	112,460
	Maximum	6896	116,640

**Table 5:** Summary of the distance and the intensity measurements for the target watering can and the black matte paper wall.

Mode		Legacy	OCDMA
Distance (m)	Minimum	0.94389	0.96274
	Maximum	1.5639	1.5309
Intensity	Minimum	31,238	555,860
	Maximum	840,556	14,242,574
